# Antimicrobial Resistance and Virulence-Associated Markers in *Campylobacter* Strains From Diarrheic and Non-diarrheic Humans in Poland

**DOI:** 10.3389/fmicb.2020.01799

**Published:** 2020-08-04

**Authors:** Beata Wysok, Joanna Wojtacka, Marja-Liisa Hänninen, Rauni Kivistö

**Affiliations:** ^1^Department of Veterinary Public Health, Faculty of Veterinary Medicine, University of Warmia and Mazury in Olsztyn, Olsztyn, Poland; ^2^Department of Food Hygiene and Environmental Health, Faculty of Veterinary Medicine, University of Helsinki, Helsinki, Finland

**Keywords:** *Campylobacter*, foodborne pathogen, diarrhea, antimicrobial resistance, virulence factors

## Abstract

Campylobacteriosis is one of the most common causes of bacterial gastroenteritis. However, the clinical course of the illness varies in symptoms and severity. The aim of this study was to characterize *Campylobacter jejuni* (34 isolates) and *C. coli* (9 isolates) from persons with diarrheal and non-diarrheal stools at the time of examination and fecal sampling, in Poland by using whole-genome sequencing (WGS). Multilocus sequence typing (MLST) analysis revealed a high diversity with a total of 20 sequence types (STs) among 26 *Campylobacter* isolates from diarrheic and 13 STs among 17 isolates from non-diarrheic persons. ST-50 and ST-257 were most common in both groups. The phenotypic resistance rate was 74.4% for ciprofloxacin, 67.4% for sulfamethoxazole/trimethoprim, 58.1% for amoxicillin, 48.8% for tetracycline, and 46.5% for ceftriaxone. Only single isolates were resistant to erythromycin, gentamicin, and amoxicillin/clavulanic acid. Overall genotypic resistance toward amoxicillin, fluoroquinolones, tetracyclines, and aminoglycosides was predicted to occur in 93.1, 67.4, 48.8, and 11.6% of the isolates, respectively. None of the isolates showed the presence of the *erm*(*B*) gene or mutation in 23S rRNA. Neither was variation found in the important target region in L4 and L22 ribosomal proteins. In regard to the CmeABC efflux pump, a set of variable mutations affecting the regulatory region was noted. All *Campylobacter* isolates possessed genes associated with adhesion (*cadF*, *jlpA*, *porA*, and *pebA*) and invasion (*ciaB*, *pldA*, and *flaC*). The type IV secretion system (T4SS) was found in isolates from both diarrheic (15.4%, CI 95%: 6.1–33.5%) and non-diarrheic (23.5%, CI 95%: 9.6–47.3%) persons. The rates of the presence of cytolethal distending toxin *cdtABC* gene cluster and type VI secretion system (T6SS) were higher in *Campylobacter* isolates obtained from persons with diarrhea (96.2%, CI 95%: 81.7–99.3% and 26.9%, CI 95%: 13.7–46.1%) compared to isolates from non-diarrheic persons (76.5%, CI 95%: 52.7–90.4% and 11.8%, CI 95%: 3.3–34.3%). The lack of statistically significant differences between two groups in tested virulence factors suggests that individual susceptibility of the host might play more determining role in the disease outcome than characteristics of the infecting strain.

## Introduction

Campylobacteriosis is the most commonly reported cause of human bacterial gastroenteritis. In 2018, the number of reported cases in the EU was 246,571, whereas salmonellosis and yersiniosis were confirmed, respectively, in 91,857 and 6,699 cases (European Food Safety Authority [EFSA], 2019). Poultry and poultry meat are considered as one of the main reservoirs and sources of human *Campylobacter* infections. In addition, a broad range of other animals, such as cattle and swine can harbor these bacteria in their digestive track (Wysok et al., 2015). Also, contact with dogs and cats is considered as a risk factor for human campylobacteriosis (Andrzejewska et al., 2013).

Persons infected with *Campylobacter* usually manifest the symptoms including gastroenteritis with bloody or mucoid diarrhea (Allos, 2001). Also, extragastrointestinal manifestations such as bacteremia, meningitis, and reactive arthritis may occur especially in immunocompromised individuals (Kaakoush et al., 2015). However, Allos (2001) underlined that in some patients, abdominal pain without diarrhea is the predominant feature. According to Janssen et al. (2008), in 30% of patients, campylobacteriosis does not start with diarrhea but with a prodrome of influenza virus-like symptoms such as fever, headache, dizziness, and myalgia. Both bacterial virulence factors and host susceptibility factors are thought involved in determining the disease outcome.

Multiple bacterial factors have been implicated in the pathogenesis of campylobacteriosis including adhesion, invasion, and production of certain toxins, which support *Campylobacter* to invade the host, cause disease, and evade host defense (de Oliveira et al., 2019). Identification of virulence factors involved in the pathogenesis of campylobacteriosis is crucial to better understanding the mechanisms of the infection and to identify if certain, potentially more virulent strains exist.

In most cases, the illness requires no specific antimicrobial treatment. However, in severe cases antimicrobial therapy and hospitalization may be needed (Allos, 2001; Wieczorek and Osek, 2013). The average hospitalization rate in the EU countries has been 30.5%, but in Poland, Latvia, Romania, and the United Kingdom, very high hospitalization rates from 80 to 100% have been noted (European Food Safety Authority [EFSA], 2017). Macrolides, such as erythromycin and azithromycin, are the most commonly used antimicrobial agents in the treatment of campylobacteriosis. Fluoroquinolones, tetracyclines, and aminoglycosides are also choices in the treatment (Wieczorek and Osek, 2013). However, growing antimicrobial resistance is a global issue. According to the monitoring protocol of Center for Disease Control and Prevention, *Campylobacter* isolates of human origin should be tested for resistance to antibiotics such as ciprofloxacin, erythromycin, tetracycline, and gentamicin (Center for Disease Control and Prevention [CDC], 2018). Resistance to antimicrobial agents can be tested phenotypically or predicted by sequence-based methods (Su et al., 2019).

Studies showing clonal relationship between strains are very important to trace the sources and routes of transmission and improve the robustness of epidemiological association studies (Kovanen et al., 2014). MLST is a commonly used method for analyzing the molecular epidemiology of *Campylobacter* (Dingle et al., 2001). Presently, whole-genome sequencing (WGS) is considered to be a superior and increasingly available method for molecular subtyping and comprehensive comparative genomic analyses, providing information also on the antibiotic resistance profiles and presence of virulence factors among strains (Kovanen et al., 2014, 2016; Llarena et al., 2017). Recent studies have highlighted the value of WGS in monitoring the antimicrobial resistance (AMR) patterns in *Campylobacter*. High concordance (97.5%) was shown to occur between the presence of AMR determinants in WGS data and phenotypic resistance among 528 *C. jejuni* and *C. coli* isolates from England and Wales (Painset et al., 2020). Similar results were recently reported from Latvia (Meistere et al., 2019).

In this study, we applied WGS to determine the diversity of *Campylobacter* strains obtained from humans with and without diarrhea. In order to test the hypothesis that genetic differences exist between the two groups of isolates, we studied the genotypes and presence of certain known virulence factors among the isolates. Furthermore, we compared the MIC levels for eight antimicrobial agents with genotypic resistance profiles.

## Materials and Methods

### Bacterial Strains

A total of 43 epidemiologically unrelated *Campylobacter* isolates were included in the study. The human isolates were obtained from the regional sanitary—epidemiological units and from the diagnostic microbiology laboratory strain collections from northeastern Poland from 2011 to 2013. The isolates were originally recovered from routine stool samples, and all samples were anonymized. Twenty six *Campylobacter* isolates were obtained from persons with diarrheal stools routinely examined for the presence of enteric pathogens. Seventeen isolates were acquired from persons diagnosed with symptoms like abdominal pain but without signs of loose stool up to the time of examination and fecal sampling. All fecal samples were bacteriologically examined at the request of a physician. *Campylobacter* spp. were isolated from the fecal samples using modified charcoal cefoperazone deoxycholate agar (mCCDA) (Oxoid, United Kingdom) incubated under microaerobic conditions at 41 ± 1°C for 48 h. Characteristic colonies were confirmed as belonging to the genus *Campylobacter* in accordance with ISO 10272-1,2017. All collected *Campylobacter* isolates were sent to the Department of Food Hygiene and Environmental Health of University of Helsinki for further analyses.

### DNA Isolation

The isolates were cultivated on mCCDA plates (Oxoid, United Kingdom). After incubation, typical colonies were Gram stained. DNA was extracted from pure cultures grown on nutrient blood agar microaerobically at 37°C for 24 h, using the PureLink Genomic DNA Mini Kit (Invitrogen, United States) according to the manufacturer’s instructions. Quantification was performed using the Qubit fluorometer (Life Technologies, Invitrogen, CA, United States). Prior to WGS analysis, DNA was stored at −20°C.

### Genome Sequencing, Assembly, MLST, and Phylogenetic Analyses

Whole-genome sequencing was performed at the Institute for Molecular Medicine Finland (FIMM) using Nextera DNA Flex Library Prep Kit and Illumina HiSeq or MiSeq. The raw sequence reads were deposited in GenBank under BioProject Accession number PRJNA549025.

A docker image of INNUca v.4.2.0-03, INNUENDO pipeline for quality control of reads, *de novo* assembly, and contigs quality assessment (Machado et al., 2019) was used to assemble draft genomes from the raw reads and to determine the sequence types (STs) of the *Campylobacter* isolates. Novel alleles and STs were submitted to the *Campylobacter* MLST database^1^. Prokka (Seemann, 2014) was used for annotating the genomes. Then, Roary (Page et al., 2015) was used to generate the core gene alignments and FastTree (Price et al., 2010) to infer the approximately maximum-likelihood phylogenetic tree based on the core gene alignments using the generalized time-reversible (GTR) model of nucleotide evolution. Jobs were run in parallel using GNU Parallel (Tange, 2011). The trees (rooted at midpoint) were visualized using iToL v4^2^ (Letunic and Bork, 2016). *Campylobacter* isolates obtained in this study were compared with MLST allele sequences of all *Campylobacter* isolates from different sources including poultry, swine, and cattle from Poland available at PubMLST database^3^ and from previous publications (Wieczorek et al., 2017; Wieczorek and Osek, 2018; Fiedoruk et al., 2019). A neighbor-joining tree was constructed based on the concatenated seven-locus MLST sequence and visualized with iTOL v4 (see text footnote 2) (Letunic and Bork, 2016).

### Antimicrobial Susceptibility Testing

The antimicrobial susceptibility was determined by the E-test according to the protocol of the European Committee on Antimicrobial Susceptibility Testing (EUCAST) for fastidious organisms. *C. jejuni* ATCC 33560 was used for quality control. All *Campylobacter* isolates were suspended into Brain Heart Infusion (BHI) broth to a turbidity equivalent to a 0.5 McFarland standard. Mueller–Hinton agar plates supplemented with 5% of defibrinated horse blood (Oxoid) and 20 mg/L β-NAD (Sigma Aldrich) were inoculated with the suspension prepared. The following M.I.C.Evaluator (Oxoid) strips were placed on the surface of dry plates: erythromycin (E, 0.015–256 μg/ml), gentamicin (CN, 0.015–256 μg/ml), ciprofloxacin (CIP, 0.002–32 μg/ml), amoxicillin (AML, 0.015–256 μg/ml), tetracycline (TET, 0.015–256 μg/ml), amoxicillin/clavulanic acid (AMC, 0.015–256 μg/ml), ceftriaxone (CRO, 0.002–32 μg/ml), and trimethoprim/sulfamethoxazole (SXT, 0.002–32 μg/ml). The plates were incubated at 41 ± 1°C for 24–48 h at microaerophilic atmosphere. Zones of inhibited growth for erythromycin, ciprofloxacin, and tetracycline were determined according to EUCAST breakpoints for *Campylobacter*^3^. Since the MIC breakpoints for the remaining tested antimicrobials were not specified for *Campylobacter* by EUCAST, we used the breakpoints for Enterobacteriaceae.

### Identification of Resistance and Virulence Genes

Identification of genes encoding resistance to tetracyclines, β-lactams, aminoglycosides, macrolides, and fluoroquinolones in the draft *Campylobacter* genomes was performed using both ResFinder (Zankari et al., 2012) and Comprehensive Antibiotic Resistance Database (CARD) (Jia et al., 2017) as well as the Rapid Annotation using Subsystem Technology (RAST) web server (Aziz et al., 2008; Overbeek et al., 2014). The *blaOXA*_61_-positive isolates were screened toward the single-nucleotide mutation (transversion G → T) in the promoter region of the target gene (Zeng et al., 2014). NCBI blast tools were used to compare nucleotide sequences.

The occurrence of genes associated with adherence (*cadF*, *jlpA*, *porA*, and *pebA*), invasion (*ciaB*, *flaC*, and *pldA*), cytolethal distending toxin (*cdtA*, *cdtB*, and *cdtC*), T4SS (*virB11*, *virB10*, *virB9*, *virB8*, *virB7*, *virB6*, *virB5*, *virB4*, and *virD4*) and T6SS (*tssA*, *tssB*, *tssC*, *hcp*, *tssE*, *tssF*, *tssG*, *tagH*, *vgrG*, *tssJ*, *tssK*, *tssM*, and *tssL*) were determined using RAST and NCBI BLAST searches. The sequences were compared against the reference genomes of *C. jejuni* (strain NCTC 11168) and *C. coli* (strain OR12). The presence of a gene was established using a 90% similarity threshold.

### Statistical Analysis

Statistical tests were performed using Statistica (StatSoft, version 13.3). Comparisons between the prevalence of resistance and virulence determinants among *Campylobacter* isolates from diarrheic and non-diarrheic persons were performed using the Fisher’s exact test. Statistical significance was defined as *P* < 0.05.

Epitools was used to calculate the 95% confidence intervals (CI 95%) using the Wilson method^4^.

## Results

### Genetic Diversity of the Isolates

**FIGURE 1:**
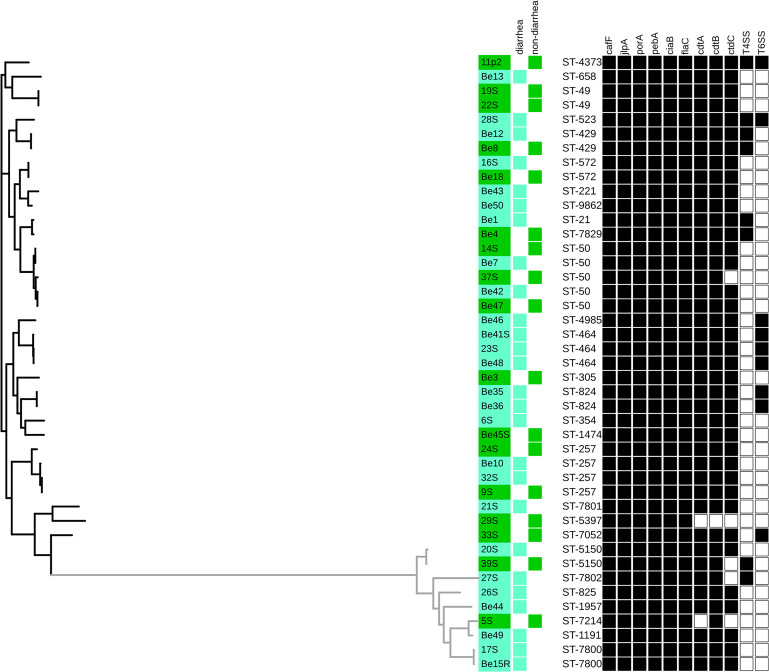
Phylogenetic analysis of *C. jejuni* and *C. coli* isolates from diarrheic and non-diarrheic persons based on their core genome alignments (246 core genes shared by ≥99% of the strains). Prevalence of determinants involved in virulence is indicated by black (present) and white (absent) squares. *C. coli* isolates form the gray cluster. Visualized in the interactive Tree of life tool (iTol).

*Campylobacter jejuni* was the predominant species found in 19 out of 26 (73.1%, CI 95%: 53.9–86.3%) diarrheic persons and 15 out of 17 (88.2%, CI 95%: 65.7–96.7%) non-diarrheic persons. The remaining isolates were identified as *C. coli*. MLST analysis revealed a total of 20 STs among 26 *Campylobacter* isolates from persons with diarrhea and 13 STs among 17 *Campylobacter* isolates from persons without diarrhea (Figure 1). The majority of STs, 12/20 among diarrheic samples and 7/13 among non-diarrheic samples, occurred only once. Out of nine STs represented by more than one isolate, the most common were ST-50 and ST-257 among diarrheic samples [noted in both in 7.7% CI 95%: 2.1–24.1%) of isolates] and among non-diarrheic samples (noted in 17.6%, CI 95%: 6.2–41.0% and 11.8%, CI 95%: 3.3–34.3% of isolates, respectively). Four novel STs were identified in diarrheic samples, ST-7801 and ST-9862 in *C. jejuni* and ST-7800 and ST-7801 in *C. coli*. Six STs were unassigned to any clonal complex (CC, analysis April 2020). The ST-21 complex (including 7 out of 43 isolates, 16.3%, CI 95%: 8.1–29.9%), ST-828 complex (6/43, 13.9%, CI 95%: 6.6–27.3%), and ST-257 complex (6/43, 13.9%, CI 95%: 6.6–27.3%) were the largest CCs. Moreover, the ST-828 complex, including 5 STs (ST-825, ST-1191, ST-1957, ST-7214, and ST-7800) showed the greatest diversity.

**FIGURE 2:**
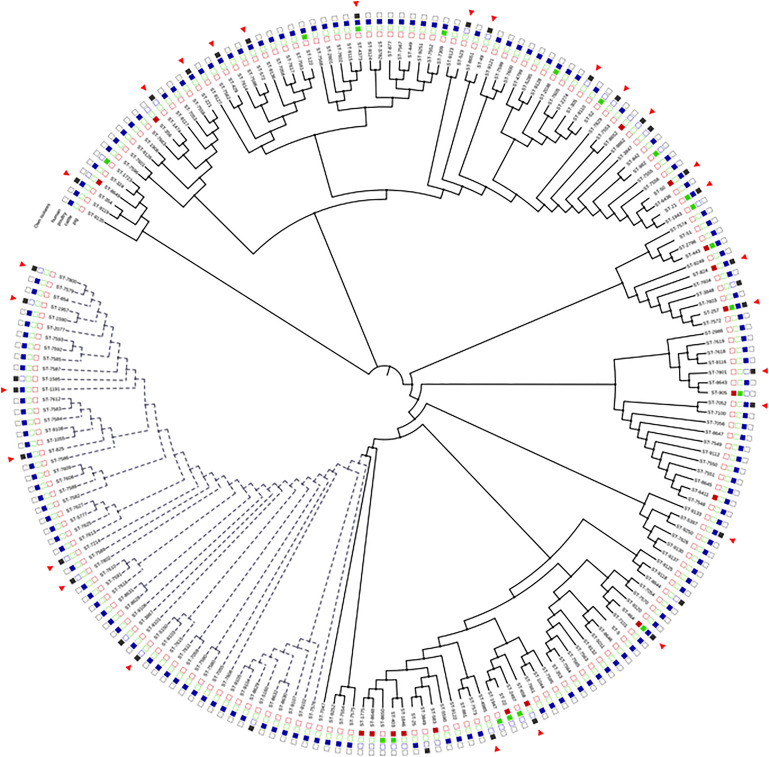
Neighbor-joining tree of concatenated MLST allele sequences among *Campylobacter* isolates originating from this study and different sources previously reported from Poland (from the PubMLST database and Wieczorek et al., 2017; Wieczorek and Osek, 2018; Fiedoruk et al., 2019). Isolates obtained in this study are marked by red triangles. Black-colored square indicates humans as source of the isolate(s), blue poultry, green cattle, and red pig. Dotted lines indicate *C. coli* isolates; other isolates are *C. jejuni*. Visualized in the interactive Tree of life tool (iTol).

Comparison of the STs of *Campylobacter* spp. isolates obtained from different sources from Poland is shown in Figure 2. Majority of the STs (57% for *C. jejuni* and 29% for *C. coli*) in our study were reported previously also in poultry isolates, and 19% and 24% of the STs for *C. jejuni* in cattle and pigs, respectively. Two of the most common STs in our study, ST-464 and ST-257, were found in all four different sources, while ST-50 was previously detected in humans, poultry, and swine, but not in cattle. Most of the STs for *C. coli* (71%) were previously undetected from other sources in Poland. Only 38% of the *C. jejuni* STs had not been previously found in other sources in Poland.

### Antimicrobial Susceptibility Testing

Overall, the highest phenotypic resistance rates were observed for ciprofloxacin (CI 95%: 59.8–85.1%), sulfamethoxazole/trimethoprim (CI 95%: 52.5–79.5%), and amoxicillin (CI 95%: 43.3–71.6%), both among isolates from diarrheic and non-diarrheic samples. Slightly lower rates of resistance to tetracycline (CI 95%: 34.6–63.2%) and ceftriaxone (CI 95%: 32.5–61.1%) were noted (Table 1). Simultaneously, only single *Campylobacter* isolates were resistant to macrolides (3 out 43, CI 95%: 2.4–18.6%), aminoglycosides (2 out of 43 isolates, CI 95%: 1.3–15.5%), and combination penicillin-type antibiotic (2 out of 43, CI 95%: 1.3–15.5%) (Supplementary Table S1).

**TABLE 1:** Comparison of phenotypic antimicrobial resistance in *Campylobacter* isolates from diarrheic and non-diarrheic samples.

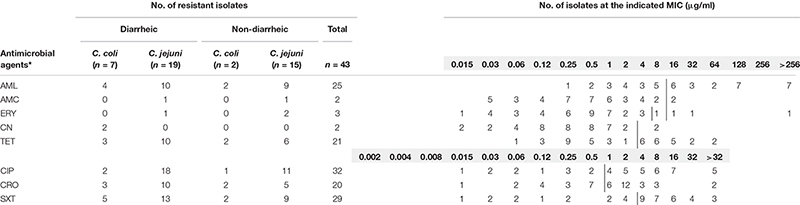

Vertical lines delineate breakpoints given by EUCAST. For erythromycin (E), separate breakpoints are given for *C. jejuni* (≥4) and *C. coli* (≥8). * Antimicrobial agents: AML, amoxicillin; AMC, amoxicillin/clavulanic; ERY, erythromycin; CN, gentamicin; TET, tetracycline; CIP, ciprofloxacin; CRO, ceftriaxone; SXT, trimethoprim/sulfamethoxazole.

Twenty three different patterns of phenotypic antimicrobial resistance were noted in this study. None of the isolates was resistant to all tested antimicrobial agents. Simultaneously, we did not observe any isolate susceptible to all studied classes of antibiotics. A total of 26 isolates (CI 95%: 45.6–73.6%) were resistant to three or more antimicrobial agents.

### Antimicrobial Resistance Genes

The presence of common antimicrobial resistance genes among *Campylobacter* isolates from diarrheic and non-diarrheic persons showed no significant differences (*P* > 0.05). The overall presence of resistance-gene profiles toward tetracyclines, fluoroquinolones, aminoglycosides, and β-lactams among human *C. jejuni* and *C. coli* isolates is shown in Supplementary Table S1.

Overall, a total of 40 isolates (CI 95%: 81.4–97.6%) were positive for one of two noted β-lactamase genes, *blaOXA*_61_ or *blaOXA*_184_. Both among isolates from humans with diarrhea and without diarrhea, the prevailing gene was the *blaOXA*_61_ noted in 37 out of 40 isolates (CI 95%: 80.1–97.4%). Simultaneously among 18/37 of *blaOXA*_61_-positive isolates, the point mutation associated with phenotypic resistance was noted and was more common in isolates originating from 10 out of 15 non-diarrheic persons (CI 95%: 41.7–84.8%) compared to 8 out of 22 isolates from diarrheic persons (CI 95%: 19.7–57.1%) (*P* = 0.0696). The less common *blaOXA*_184_-positive isolates were assigned to ST-7052, ST-7801, and ST-5397, which are closely related.

The genes associated with resistance to tetracyclines were found among 21 out of 43 (CI 95%: 34.6–63.2%) isolates, and simultaneously all isolates exhibited resistance to tetracycline. Most of the isolates carried the intact *tet(O)* gene, but in 6 isolates from diarrheal samples (assigned to ST-464, ST-5150, ST-7802, and ST-523) and in 3 isolates of non-diarrheal samples (assigned to ST-4373, ST-5397, and ST-5150), the mosaic gene *tet(O/32/O)* was present.

Resistance to fluoroquinolones associated with mutations in the *gyrA* gene was found among 29 out of 43 of the isolates (67.4%, CI 95%: 52.5–79.5%). The Thr-86-Ile mutation in *gyrA* gene was present only in *C. jejuni* isolates. The phenotypic resistance to ciprofloxacin was confirmed in 32 out of 43 isolates (74.4%, CI 95%: 59.8–85.1%), and aminoglycoside resistance, conferred by *aadE-Cc* and *aph(3’)-III* gene, was noted in 11.6% (CI 95%: 5.1–24.5%) of *Campylobacter* isolates. The *aadE-Cc* gene was found only among 2/7 *C. coli* isolates from diarrhea samples, whereas the *aph(3’)-III* gene was present both in *C. jejuni* isolates from 1 out of 19 diarrheic and 2 out of 5 non-diarrheic persons.

A high level of concordance was observed between phenotypic and genotypic resistance for gentamicin and presence of the *aadE_Cc* gene (100%), tetracycline and *tet(O)* or *tet(O/32/O)* (100%), and ciprofloxacin and T86I mutation in the *gyrA* gene (93%) (Table 2).

**TABLE 2:** Comparison between phenotypic and genotypic resistance in *Campylobacter* isolates.

	Phenotypic resistance	Genotypic resistance	Concordance (%)
Ciprofloxacin resistance versus mutation T86I *gyrA*	R (*n* = 32)	R (*n* = 29)	93
Tetracycline versus *tet*(O) or *tet(O/32/O)*	R (*n* = 21)	R (*n* = 21)	100
Gentamicin versus *aadE-Cc*	R (*n* = 2)	R (*n* = 2)	100
Amoxicillin versus *blaOXA61*	R (*n* = 23)	R (*n* = 37)	67.4
*Including:* Isolates with noted mutation in *blaOXA61*	R (*n* = 12)	R (*n* = 17)	88.4
Amoxicillin versus *blaOXA184*	R (*n* = 2)	R (*n* = 3)	97.7

To determine the erythromycin resistance, the isolates were screened for the presence of *23S rRNA*, *rplV*, *rplD*, and *cmeRABC* locus mutations and for the presence of the *erm(B)* gene. None of the isolates tested showed the presence of the *erm(B)* gene or mutation in *23S rRNA.* Overall, 35 isolates possessed substitution in the ribosomal protein L4 (V196A) and 3 isolates possessed substitution T91K. In regard to ribosomal protein L22 among 13 isolates, the following substitutions were noted: S13P, G74A, A103V, T109A, A111E, A114T, T119A, A121V, and A136T. Among 11 out of 43 (25.6%, CI 95%: 14.9–40.2%), the mutation in the intergenic region between *cmeR* and *cmeA* was observed including single substitution or single deletion. Further analysis of the *cmeR* gene revealed that 11 out of 26 (42.3%, CI 95%: 25.5–61.1%) of isolates from diarrheic persons and 11 out of 17 (64.7%, CI 95%: 41.3–82.7%) of isolates from non-diarrheic persons showed the presence of mutation, resulting in amino acid changes. The most common mutation noted in isolates from both diarrheic and non-diarrheic samples were G144D, P183R, and S207G.

Ten different patterns were noted among antimicrobial resistance gene content (Table 1). None of the isolates was found to be genetically susceptible to all antimicrobials tested. Multiresistant genotypes to at least three different antimicrobial classes were found among 12 out of 26 of *Campylobacter* isolates from diarrheic and among 6 out of 17 from non-diarrheic samples.

### Virulence Factors

All *C. jejuni* and *C. coli* isolates obtained from diarrheic and non-diarrheic persons possessed the genes associated with adherence (*cadF*, *jlpA*, *porA*, and *pebA*) and invasion (*ciaB*, *flaC*, and *pldA*) (Figure 1).

The *cdt* gene cluster was confirmed to be more common among *Campylobacter* spp. isolates from diarrheic samples (25/26, 96.2%, CI 95%: 81.1–99.3%) than among isolates from non-diarrheic samples (13/17, 76.5%, CI 95%: 52.7–90.4%) (*P* = 0.071). Out of three genes associated with cytotoxin production, the lowest prevalence was noted for the *cdtC* gene (38/43, 88.4%, CI 95%: 75.5–94.9%) compared to levels observed for *cdtB* (42/43, 97.7%, CI 95%: 87.9–99.6%) and *cdtA* (41/43, 95.5%, CI 95%: 84.5–98.7%) genes (Figure 1).

The prevalence of type IV secretion system genes (T4SS) was slightly higher in *Campylobacter* isolates from non-diarrheic persons (23.5%, CI 95%: 9.6–47.3%) than in isolates from diarrheic persons (15.4%, CI 95%: 6.1–33.5%) (*P* > 0.05). Three out of four isolates from diarrheic samples had all tested genes (*virB11*, *virB10*, *virB9*, *virB8*, *virB7*, *virB6*, *virB5*, *virB4*, and *virD4*), and all were obtained from bloody diarrhea. Isolate Be12 was positive for 7 out of 9 tested genes (*virB11*, *virB10*, *virB9*, *virB8*, *virB6*, *virB4*, and *virD4*). Two out of 4 isolates obtained from non-diarrheic persons (isolates 11p2 and Be4) were positive for all T4SS cluster genes. Isolates Be8 and 39S were missing *virB5* + *virB7* genes and *virD4* gene, respectively.

The occurrence of T6SS-positive isolates among *C. jejuni* was higher for diarrheic isolates 7/26 (26.9%, CI 95%: 13.7–46.1%) than non-diarrheic isolates 2/17 (11.8%, CI 95%: 3.3–34.3%) (*P* = 0.2491). 3 out of 7 *Campylobacter* isolates positive for T6SS originating from diarrheal persons were obtained from stools with the presence of blood. However, none of the isolates from both diarrheic and non-diarrheic persons contained the entire T6SS locus. T6SS-positive isolates showed the presence of nine (*tssJ*, *tssB*, *tssC*, *hcp*, *tssE*, *tssA*, *tssG*, *tagH*, and *tssK*) out of the 13 genes described for the complete locus (Silverman et al., 2012). Among T6SS-positive isolates from diarrheic samples, five out of seven belonged to either ST-464 or ST-824.

## Discussion

Most *Campylobacter* strains described in various studies were isolated from persons with diarrhea as the most common symptom (Kovanen et al., 2014; Jelovcan et al., 2016; Ramonaite et al., 2017). However, some infected people may have symptoms, e.g., abdominal pain without watery or bloody diarrhea (Allos, 2001). Based on WGS, we compared *Campylobacter jejuni* and *C. coli* isolates from diarrheic and non-diarrheic persons, who visited their physician to seek medical advice, to better understand the difference between strains of *Campylobacter* and associated disease outcome. The division of study groups into diarrheic and non-diarrheic humans was based on the information given by their physician. The results also allow a better understanding of the population structure and antimicrobial resistance profiles of *Campylobacter* isolates.

*Campylobacter jejuni* was the most prevalent species, among samples from persons both with diarrhea (73.1%) and without diarrhea (88.2%). The remaining isolates were *C. coli*. According to Kaakoush et al. (2015), gastroenteritis caused by *Campylobacter* species is less frequently induced by *C. coli* than *C. jejuni* but can be as high as 25% of cases. These findings are in accordance with our data, and similar results were recently noted not only in Poland (Szczepańska et al., 2017) but also in Austria (Jelovcan et al., 2016) and Germany (Gurtler et al., 2005).

In persons with diarrhea, both secretory (73.1%) and bloody (26.9%) stools were observed. A similar rate of samples with the presence of blood was observed by Tracz et al. (2005) and Quetz et al. (2012) at levels of 27% and 30%, respectively. According to Janssen et al. (2008), adherence of *Campylobacter* to the intestine and the production of toxins, which alter the fluid resorption capacity of the intestine, resulted in secretory diarrhea, while bacterial invasion and replication within the intestinal mucosa were accompanied by an inflammatory response resulting in blood-containing inflammatory diarrhea.

*Campylobacter* isolates showed considerable variation in their sequence types, irrespective of the species. In total, 26 *Campylobacter* isolates from diarrheic persons were assigned to 20 STs, while out of 17 isolates from non-diarrheic persons 13 STs were found. Genetic diversity of human *Campylobacter* isolates has been reported, in many geographic regions, i.e., in Lithuania (Ramonaite et al., 2017), in Austria (Jelovcan et al., 2016), in Finland (Kovanen et al., 2014), and in Denmark (Joensen et al., 2018). Moreover, in the present study, the majority of STs (67.9%) were represented by a single isolate, underlining the non-epidemic background of the *Campylobacter* infections. In our study, ST-50 and ST-257 were the most common. These STs have been described as prevailing both in humans (Mossong et al., 2016; Elhadidy et al., 2018) and in poultry (Dearlove et al., 2016; Wieczorek et al., 2017) in different geographical regions. Majority of the STs (57% for *C. jejuni* and 29% for *C. coli*) in our study, however, have been reported previously not only in poultry isolates but also from cattle and pigs. For example, ST-429, noted in the current study both from diarrheic and non-diarrheic persons, belongs to CC-48, which has been frequently connected with cattle (An et al., 2018; Wieczorek and Osek, 2018). The most common STs in our study, ST-464, ST-257, and ST-50, have been identified in multiple sources in Poland and elsewhere, supporting the evidence that the most successful strains are able to colonize several host species and thus are also more widespread in the food chain and the environment.

Resistance against β-lactams is high among *Campylobacte*r isolates, and majority of them, irrespective of geographical regions, produce beta-lactamases (Wieczorek and Osek, 2013; Reddy and Zishiri, 2017; Redondo et al., 2019). Similarly, in this study the most common antimicrobial resistance determinants were beta-lactamase genes, *blaOXA*_61_ and *blaOXA*_184_. However, Griggs et al. (2009) underlined that the role of β-lactamase genes in the mechanism of resistance to ampicillin in campylobacters are not yet clear, and the production of β-lactamase is not always associated with resistance to β-lactams.

In this study, 15 out of 40 (CI 95%: 37.5–52.9%) isolates possessing the *blaOXA* gene were sensitive to amoxicillin. Similar results were noted by Aslantaş (2017) who confirmed susceptibility to ampicillin in 14.9% of *C. jejuni* and 32.5% of *C. coli* isolates that harbored this marker. Zeng et al. (2014) underlined that some *C. jejuni* strains that carry the β-lactamase genes *blaOXA*_61_ are still susceptible to β-lactams with undetected β-lactamase activity, suggesting that a point mutation (G→T transversion) upstream *blaOXA*_61_ conferred high-level ampicillin resistance in *Campylobacter* strains. In our study, altogether 70.6% (12 out of 17 isolates) of *Campylobacter* isolates with confirmed presence of the single-nucleotide mutation upstream of the *blaOXA*_61_ gene were resistant to amoxicillin. The observed resistance levels were significantly higher among isolates with confirmed mutation (83.3% of isolates showed the MIC values ≥ 128 μg/ml) compared to isolates without the mutation (30.8% of isolates showed the MIC values ≥ 128 μg/ml). Interestingly the combination of penicillin-type antibiotic (AMP, amoxicillin/clavulanic) was highly effective against *Campylobacter* (resistance exhibited by 2 out 43 isolates, CI 95%: 1.3–15.5%). No resistance to amoxicillin–clavulanic acid was detected by Post et al. (2017) in Belgium and Wallace et al. (2019) in Australia in clinical isolates. Also, the study conducted by Schiaffino et al. (2019) provides compelling evidence to propose amoxicillin/clavulanic acid as an effective treatment for campylobacteriosis.

The fluoroquinolones, as broad-spectrum antimicrobials, are frequently used especially in treatment of undiagnosed cases of diarrhea (Sproston et al., 2018). In this study, high numbers of isolates with *gyrA T86I* mutation associated with fluoroquinolone resistance occurred in isolates from both diarrheic and non-diarrheic samples (69.2% and 64.7% of isolates, respectively). This finding is in accordance with other Polish data published by Rożynek et al. (2008), who reported this mutation in 59.2% of human *Campylobacter* isolates. Also, recently published data from Poland presented by Szczepańska et al. (2017) and Wieczorek et al. (2018) showed a rising trend in resistance to these antibacterial agents. Furthermore, we noted the significantly marked correlation between phenotypic and genotypic resistance to fluroquinolones. Of 32 isolates exhibiting the resistance to ciprofloxacin, 29 (90.6%, CI 95%: 75.8–96.8%) possessed T86I mutation in the *gyrA* gene. The 3 isolates lacking the Thr-86-Ile amino acid substitution showed low-level resistance to ciprofloxacin (1 μg/ml), and all belonged to *C. coli*. These isolates exhibited other amino acid substitutions at codon 22, 237, or 285, which also occurred among isolates susceptible for CIP; therefore, their role in resistance is unlikely to be important. Charvalos et al. (1996) described other mechanisms of resistance to quinolones including decreased outer-membrane permeability and efflux systems, which may contribute to the phenotypic resistance observed in strains, where no mutations leading to amino acid changes in *gyrA* were observed. Similarly, a high level of correlation was observed between genotypic and phenotypic resistance against tetracyclines. Altogether, the *tet(O)* gene or a mosaic gene *tet(O/32/O)* was detected in 50% of the isolates from diarrheic persons and in 47.1% of isolates from non-diarrheic persons and all of these isolates exhibited phenotypic resistance to tetracycline. Also, data presented by Reddy and Zishiri (2017) revealed a wide distribution of the *tet(O)* gene in 64% of human clinical isolates. The studies conducted by Wieczorek et al. (2018) in Poland showed high phenotypic resistance to tetracyclines among human isolates at the level of 70.3%. Lopes et al. (2019) showed the prevalence of mosaic form of the *tet(O)* gene among *Campylobacter* isolates belonging to CC-464 in the United Kingdom. Also in this study, the mosaic gene *tet(O/32/O)* was noted in all *C. jejuni* isolates belonging to ST-464, indicating a wider dispersion of this resistant genotype in Europe. Our findings are in accordance with previously presented data showing high resistance rates to tetracyclines and fluoroquinolones (Szczepańska et al., 2017; Wieczorek et al., 2018), rendering these antimicrobials not useful in treatment of campylobacteriosis.

Only a few *Campylobacter* isolates possessed genes associated with aminoglycoside (e.g., gentamicin or streptomycin) resistance (approximately 11% had *aph(3’)-III* or *aadE-Cc* genes). However, previous studies have shown that the prediction of resistance against aminoglycosides is not always straightforward from WGS data using currently available tools (Meistere et al., 2019). In this study, the obtained phenotypic rate of resistance to gentamicin was slightly lower compared to the genotypic rate, confirmed in 2 out of 43 isolates (CI 95%: 1.3–15.5%). All gentamicin-resistant isolates possessed the *aadE_Cc* gene. Three isolates with the *aph(3’)-III* gene were susceptible to gentamicin. Generally, the distribution of *aphA* genes in clinical *Campylobacter* isolates confers resistance to kanamycin (Cameron and Gaynor, 2014). Also in previous studies, mostly low levels of resistance to this class of antimicrobials, using gentamicin as a model, were shown (Rożynek et al., 2008; Szczepańska et al., 2017).

In our study, none of the *Campylobacter* isolates, irrespective of the species, displayed a mutation in the 23S rRNA gene, resulting in macrolide resistance. Similarly, in previous studies conducted in Poland, very low resistance to macrolides was detected among human *Campylobacter* isolates (Rożynek et al., 2008; Szczepańska et al., 2017). According to Luangtongkum et al. (2009), several modifications in the ribosomal proteins L4 and L22 are associated with low- to intermediate-level macrolide resistance in *Campylobacter.* Mutations in the large loop of the L4 protein (residues 55 to 77) and the L22 protein (residues 78 to 98) have been confirmed to be associated with macrolide resistance in various bacteria (Zhou et al., 2016). In this study, no variations were found in these regions. The ribosomal RNA methylase gene *ermB* represents a major mechanism for macrolide resistance (Zhou et al., 2016); however, this marker was not reported in any of the isolates. Wieczorek and Osek (2013) draw attention to the presence of CmeABC multidrug efflux pump, which is the major efflux mechanism causing antimicrobial resistance to several antimicrobials including fluoroquinolones and macrolides. Expression of *cmeABC* operon is controlled by a transcriptional repressor named CmeR (Lin et al., 2005). Mutations observed in the *cmeR* gene or in the inverted repeat (IR) region of the efflux operon result in enhanced resistance to different groups of antimicrobials agents. In this study, a mutation in the *cmeR* gene was noted in 51.2% isolates, while 25.6% were positive for mutation in the intergenic region between *cmeR* and *cmeA* genes. Interestingly, the antimicrobial susceptibility testing performed in this study revealed the occurrence of resistance to erythromycin in 3 out of 43 tested isolates (CI 95%: 2.4–18.6%) and all these isolates possessed both three or more mutations in the *cmeR* gene resulting in changes of aa and mutation in the IR region. Interestingly, isolate Be3 with the highest resistance to ERY (>256 μg/ml) did not have the complete sequence for transcriptional regulatory gene *cmeR* (633 bp). A deletion of 122 bp was noted, resulting in a premature stop codon in the gene, encoding a truncated 176-amino acid product instead of the full 211-amino acid protein.

In this study, high rates of resistance were also found in ceftriaxone and trimethoprim/sulfamethoxazole, at the levels 46.5% (CI 95%: 32.5–61.1%) and 67.4% (CI 95%: 52.5–79.5%), respectively. Emonet et al. (2017) suggest that ceftriaxone is often prescribed empirically for patients hospitalized with abdominal pain and fever. However, in many studies this antimicrobial agent is not recommended for treatment of campylobacterisosis (Emonet et al., 2017; Schiaffino et al., 2019). Similarly, trimethoprim/sulfamethoxazole is not recommended to be used in routine treatment of campylobacteriosis. Studies in Taiwan (Chang et al., 2017) or Thailand (Mason et al., 2017) show high incidence of SXT resistance in *Campylobacter*, suggesting that this type of resistance is actual if these antimicrobials are used. Simultaneously, we noted that the majority of the isolates exhibiting resistance to ceftriaxone (65%, CI 95%: 43.3–81.9%) and trimethoprim/sulfamethoxazole (68.9%, CI 95%: 50.8–82.7%) possessed mutations in the *cmeR* gene or in the inverted repeat region, previously shown to result in the overexpression of CmeABC and enhanced resistance to multiple antibiotics (Lin et al., 2005). Multidrug resistance (MDR), defined as resistance to three or more classes of antimicrobial agents, is an emerging problem among pathogenic bacteria (Schwarz et al., 2010). We found a relatively high number of *C. jejuni* isolates which harbor resistance genes against at least three antimicrobial classes (38.2% of strains). In addition, three *C. jejuni* isolates had a combination of four genes associated with resistance (*gyrA*, *tet(O)*, *blaOXA*, and *aph*) increasing the total percentage of multiresistant isolates up to 47.1%. When considering the phenotypic resistance, MDR was noted in 55.8% of the isolates. Simultaneously, data presented in different geographical regions showed high and extremely high prevalence of MDR among *Campylobacter* isolates (Schiaffino et al., 2019). Further analysis revealed that all isolates assigned to ST-257, ST-429, ST-464, ST-572, and ST-824 were associated with multiresistance. Especially, ST-464 seems to have acquired multiresistance all over Europe. Among *Campylobacter* strains assigned to ST-464, the multidrug resistance to ciprofloxacin, nalidixic acid, tetracycline ciprofloxacin, tetracycline, and ceftriaxone was confirmed in the studies conducted, e.g., by Cha et al. (2016) in United States and Aksomaitiene et al. (2019) in Lithuania. Moreover, Cha et al. (2016) underlined the need to enhance surveillance efforts and identify factors associated with global spread of this resistant sequence type, especially because the majority of the ST-464 isolates were obtained from individuals in United States with a history of foreign travel. Interestingly, this sequence type was identified only among samples from diarrheic persons.

We also assessed the differences in the prevalence of virulence determinants. In pathogenesis, adhesion is a crucial step prior to invasion and secretion of toxins (Letourneau et al., 2011). All *Campylobacter* isolates from humans both with and without diarrhea were shown to have the genes encoding proteins involved in adhesion (*cadF*, *porA*, *jlpA*, and *pebA*), and invasion (*ciaB*, *pldA*, and *flaC*). These findings are in accordance with studies conducted by Biswas et al. (2011) in Canada and Wieczorek et al. (2018) in Poland, showing high prevalence of these genes among clinical samples. Thus, the ability of *Campylobacter* isolates to adhere to and invade epithelial cells seems to be common.

Another important virulence factor of *Campylobacter* spp. is the ability to produce toxins. The best-characterized toxin attributed to *Campylobacter* is cytolethal distending toxin (CDT) encoded by three adjacent genes *cdtA*, *cdtB*, and *cdtC* (Bang et al., 2003). Many studies have shown that the *cdt* gene cluster is common in human (Redondo et al., 2019), poultry (Wieczorek et al., 2018), and cattle and swine isolates (Wysok et al., 2015). Overall, in this study, the *cdtABC* gene complex was noted in 96.2% of *Campylobacter* isolates from persons with diarrhea. In turn, the prevalence of the complete *cdtABC* gene cluster was much lower (76.5%) among *Campylobacter* isolates from persons without diarrhea.

*Campylobacter* isolates have two known secretion systems—type IV secretion system (T4SS) and type VI secretion system (T6SS). The T4SS is encoded by plasmid pVir, important for both adherence and invasion of *Campylobacter* in intestinal epithelial cells (Tracz et al., 2005). In our study, T4SS was confirmed to be present in both *Campylobacter* isolates from diarrheic samples (15.4%) and non-diarrheic samples (23.5%), suggesting higher occurrence of the pVir plasmid than noted in some other geographical regions, for example, 3% in the Netherlands (Louwen et al., 2006), 10% in Thailand (Bacon et al., 2000), and 17% in Canada (Tracz et al., 2005). The significant role of the pVir plasmid in the development of symptoms observed during *Campylobacter* infection was underlined by Tracz et al. (2005). These authors noted that patients infected with a pVir-positive *Campylobacter* strain were more likely to produce bloody stool than those infected with a pVir-negative strain. Louwen et al. (2006), however, reported the absence of an association with the pVir plasmid in patients infected with *Campylobacter* who developed bloody diarrhea.

T6SS has also been shown to be an important determinant in adhesion and invasion, and it is active in contact-dependent transfer of DNA and proteins from bacteria to host cells (Chen et al., 2019). Controversial results have been presented also on the association of T6SS with the severity of symptoms. Harrison et al. (2014) reported that patients infected with T6SS-positive *Campylobacter* experienced bloody diarrhea more frequently than those infected with T6SS-negative *Campylobacter*. Moreover, these authors noted diverging prevalence rates of this determinant in different geographical regions, 2.6% in the United Kingdom, 15.4% in Pakistan, 33.3% in Thailand, and 60.6% in Vietnam. In our study, this locus was found more often in diarrheic samples (26.9%). However, none of the *Campylobacter* isolates possessed the entire T6SS cluster (13 ORFs). We noted lack of *tssL* and *tssM* genes encoding inner membrane proteins and lack of the *tssI* gene encoding VgrG protein that functions as a puncturing device toward the targeted cells (Silverman et al., 2012). However, all T6SS-positive *Campylobacter* isolates possessed the *hcp* gene highlighted by Corcionivoschi et al. (2015) as an indicative component of a functional T6SS in *C. jejuni*.

## Conclusion

Whole-genome sequencing analysis showed high ST genotype diversity of *Campylobacter* isolates from both diarrheic and non-diarrheic samples. Most common STs (ST-50 and ST-257) occurred in both groups. Nearly half of the isolates had both a resistant genotype and phenotype to at least two antimicrobial agents, and multidrug resistance was also common among the isolates. Especially interesting was ST-464 with resistance genes to tetracycline, fluoroquinolone, and beta-lactamase. This resistant genotype seems to be common all over Europe. We observed a higher prevalence of CDT and T6SS among isolates from diarrheic persons, yet further studies are needed to determine their impact on the outcome of *Campylobacter* infections in more detail.

## Data Availability Statement

The raw sequence reads produced in this study were deposited in GenBank under BioProject Accession Number PRJNA549025.

## Author Contributions

M-LH initiated and supervised the study. RK and BW designed the research and wrote the manuscript. BW analyzed the data. JW performed the statistical analysis. All authors revised the manuscript and approved the final version for submission.

## Conflict of Interest

The authors declare that the research was conducted in the absence of any commercial or financial relationships that could be construed as a potential conflict of interest.

## Footnotes

1 https://pubMLST.org/campylobacter/

2 http://itol.embl.de

3 https://eucast.org/clinical_breakpoints/

4 https://epitools.ausvet.com.au/ciproportion
